# Acute respiratory failure due to hemothorax after posterior correction surgery for adolescent idiopathic scoliosis: a case report

**DOI:** 10.1186/1471-2474-14-132

**Published:** 2013-04-11

**Authors:** Yoji Ogura, Kota Watanabe, Naobumi Hosogane, Yoshiaki Toyama, Morio Matsumoto

**Affiliations:** 1 Department of Orthopaedic Surgery, Keio University School of Medicine, Tokyo, Japan; 2 Department of Advanced Treatment for Spine and Spinal Cord Disorders, Keio University School of Medicine, Tokyo, Japan

## Abstract

**Background:**

Although posterior correction and fusion surgery using pedicle screws carries the risk of vascular injury, a massive postoperative hemothorax in a patient with adolescent idiopathic scoliosis (AIS) is quite rare. We here report a case of a 12-year-old girl with AIS who developed a massive postoperative hemothorax.

**Case presentation:**

The patient had a double thoracic curve with Cobb angles of 63° at T2-7 and 54° at T7-12. Posterior correction and fusion surgery was performed using a segmental pedicle screw construct placed between T2 and T12. Although the patient's respiration was stable during the surgery, 20 minutes after removing the trachea tube, the patient’s pulse oximetry oxygen saturation suddenly decreased to 80%. A contrast CT scan showed a massive left hemothorax, and a drainage tube was quickly inserted into the chest. The patient was re-intubated and a positive end-expiratory pressure of 5 cmH_2_O applied, which successfully stopped the bleeding. The patient was extubated 4 days after surgery without incident. Based on contrast CT scans, it was suspected that the hemothorax was caused by damage to the intercostal arteries or branches during pedicle probing on the concave side of the upper thoracic curve. Extensive post-surgical blood tests, echograms, and CT and MRI radiographs did not detect coagulopathy, pulmonary or vascular malformation, or any other possible causative factors.

**Conclusion:**

This case underscores the potential risk of massive hemothorax related to thoracic pedicle screw placement, and illustrates that for this serious complication, respiratory management with positive airway pressure, along with a chest drainage tube, can be an effective treatment option.

## Background

Hemothorax is usually related to chest trauma or to medical procedures such as central venous line insertion, thoracentesis, pleural biopsy, or intrathoracic surgeries that can injure lung parenchyma or intrathoracic vessels such as the intercostal or internal mammary arteries and their branches [1,2]. Postoperative hemothorax has been reported as a complication associated with anterior procedures and posterior thoracoplasty [1,3] in correction and fusion surgeries for scoliosis. The incidence of hemothorax in anterior scoliosis surgery is reported to be 1–2.2% [2-4].

Hemothorax may be caused by surgical procedures such as releasing the pleurae from the vertebrae, or the removal and curettage of intervertebral discs and cartilage end plates. The placing of pedicle screws in the course of posterior correction and fusion surgery carries a risk of vascular injury. However, the occurrence of hemothorax associated with corrective surgery is as low as 0.1% [5-7] in patients with adolescent idiopathic scoliosis (AIS). A review article of the Scoliosis Research Society morbidity and mortality database also showed pulmonary complication, which included complication other than hemothorax, was 0.9% [8]. All previously reported incidents of postoperative hemothorax were treated by observation without additional intervention.

Here we report the case of a patient with AIS who developed a massive hemothorax after posterior correction and fusion surgery using a pedicle screw construct. The hemothorax induced acute respiratory failure and required the insertion of a chest drainage tube.

## Case presentation

A 12-year-old girl with progressive trunk deformity was referred to our department. She had been diagnosed with AIS at 11 years old, and had started brace treatment. A full time underarm type brace was applied before surgery. The Cobb angles before brace treatment were 36° at T2-7 and 35° at T7-12. The curves were corrected to 31° and 27° with the correction rate of 15% and 22%, respectively after application of the brace. Because the deformity progressed in spite of the brace treatment, she was referred to our department to undergo surgical treatment. Physical examination found a prominent rib hump on the right side, and her right shoulder was slightly elevated. There were no abnormalities in her extremities or neurological examination. The patient weighed 31.8 kg (−1.20 SD), with a height of 132.5 cm (−2.37 SD).

**Figure 1:**
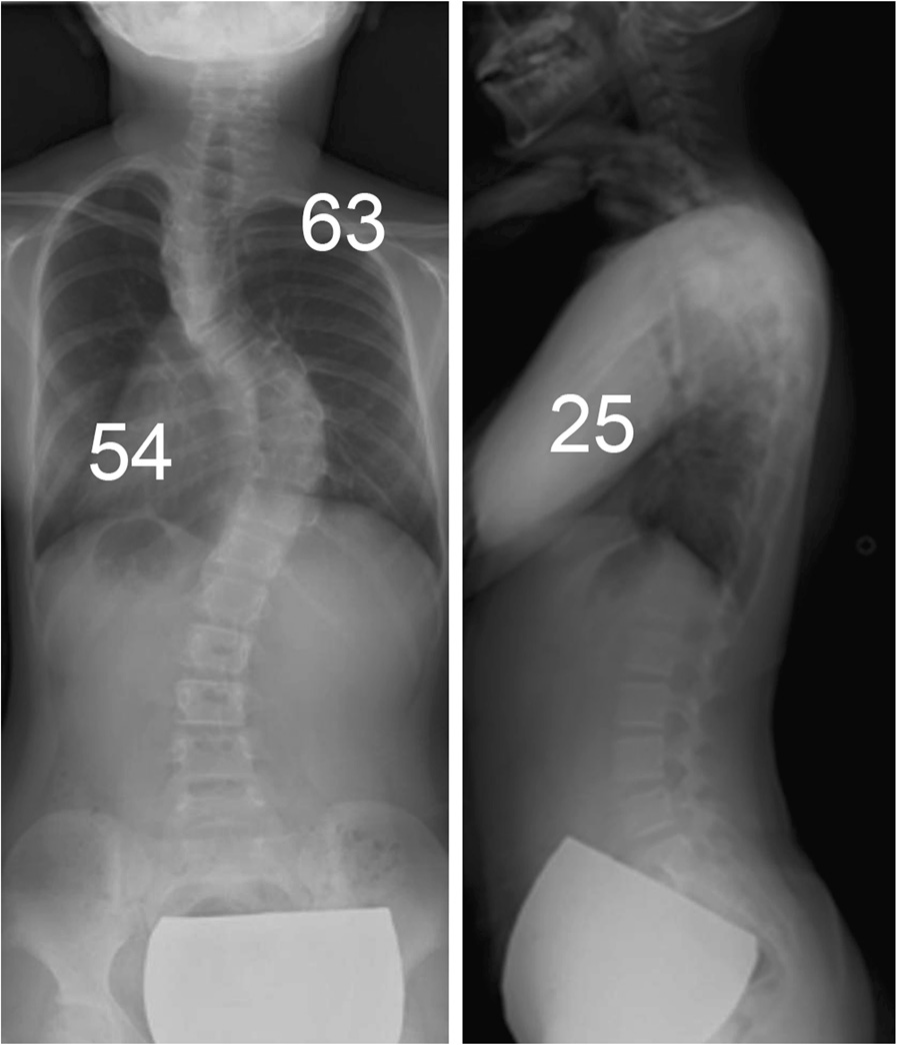
**Standing x-rays.** Standing x-rays show scoliosis of 63° at T2-7 and 54° at T7-12, and kyphosis of 25° at T5-12.

Radiographs showed scoliosis of 63° at T2-7 and 54° at T7-12, and kyphosis of 25° at T5-12 (Figure 1). Supine side-bending radiographs showed scoliosis of 50° at T2-7 and 35° at T7-12, and the curve flexibilities were 21% and 35%, respectively. The Lenke classification was type 2AN. Three-dimensional CT images showed no congenital deformity of the thoracic or lumbar spine. The mean pedicle width between T2-12 was 3.4 ± 1.3 mm (1.5-5.2 mm) on the right side, and 3.5 ± 0.9 mm (2.1-4.8 mm) on the left side. The narrowest pedicle width was 1.5 mm at T3 and T4 on the right side, and 2.1 mm at T8 on the left side. Laboratory data showed no coagulation disorders (PT, 10.6 sec; APTT, 34.4 sec), and a pulmonary function test revealed normal lung function (%VC, 84%; %FEV_1.0_, 93%).

**Figure 2:**
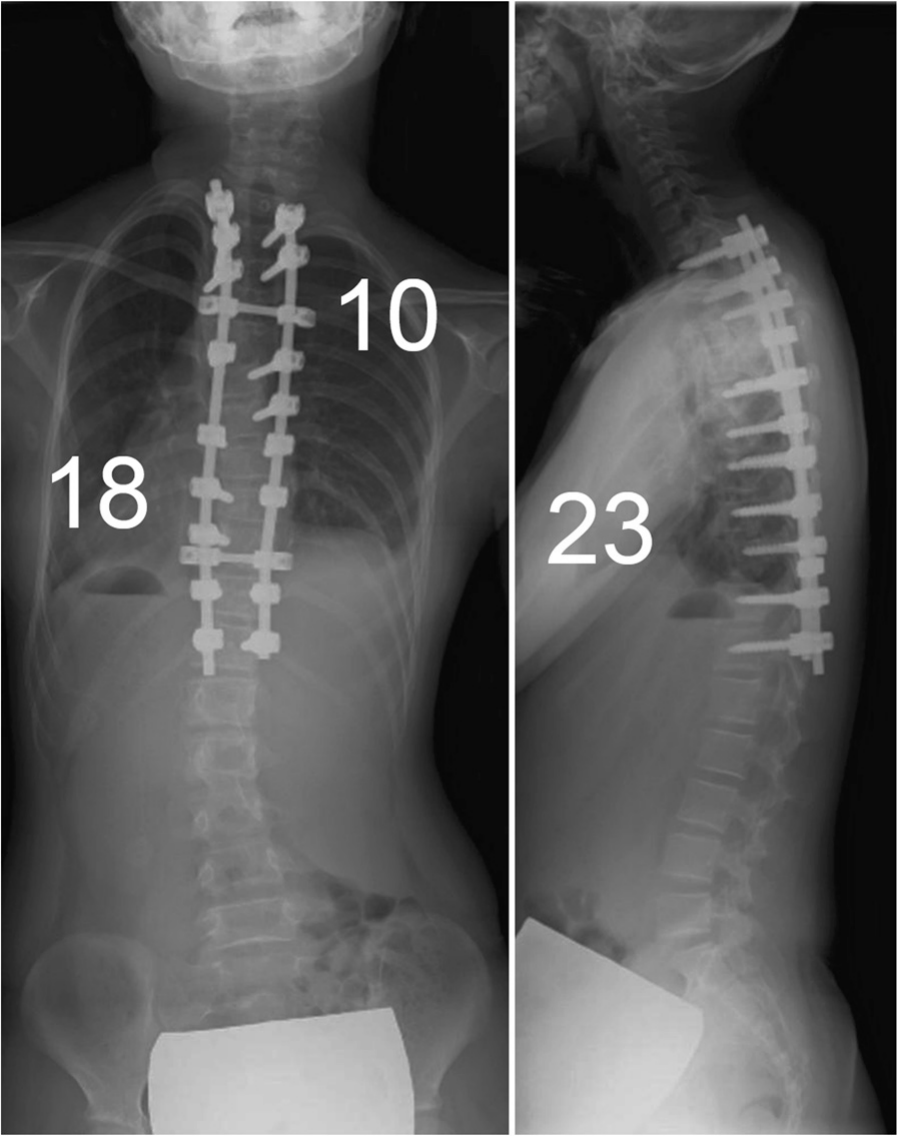
**Postoperative x-rays.** Postoperative x-rays show that the scoliosis was surgically corrected to 10° and 18°, with correction rates of 84% and 67%, respectively.

The patient underwent posterior correction and fusion surgery with a segmental pedicle screw construct at T2-12. After the posterior elements of the thoracic spine were meticulously exposed, pedicle screws were placed segmentally using a ball-tip probe technique [9]. No obvious pedicle perforation was noted during screw placements. Ponte osteotomies were performed between T3 and T6, since the proximal curve had low flexibility. The curves were corrected with a rod applied on the concave side of the main thoracic curve and in situ bending of the rod followed by a graft of local bone. The surgery took 184 minutes, with an estimated blood loss of 400 ml. The scoliosis was corrected to 10° and 18°, with correction rates of 84% and 67%, respectively (Figure 2).

**Figure 3:**
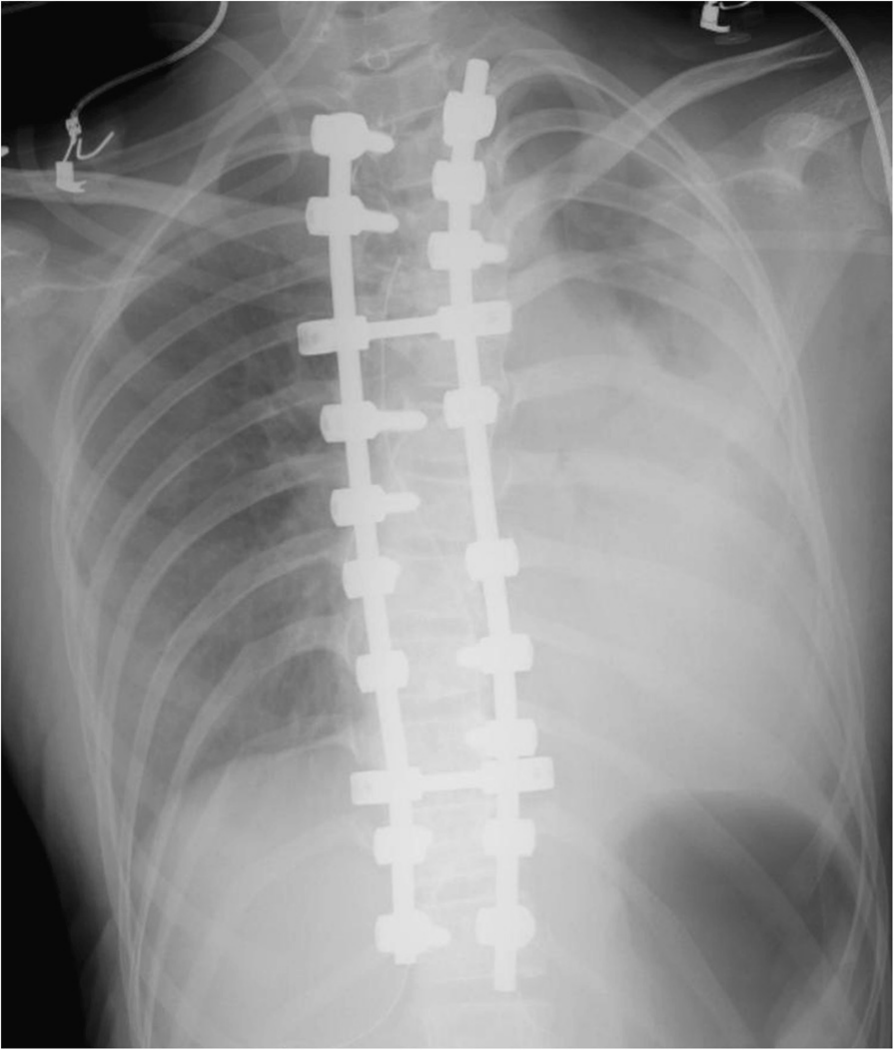
**A chest x-ray taken 20 minutes after extubation.** A chest x-ray film taken twenty minutes after extubation shows a massive hemothorax in the left lung field.

**Figure 4:**
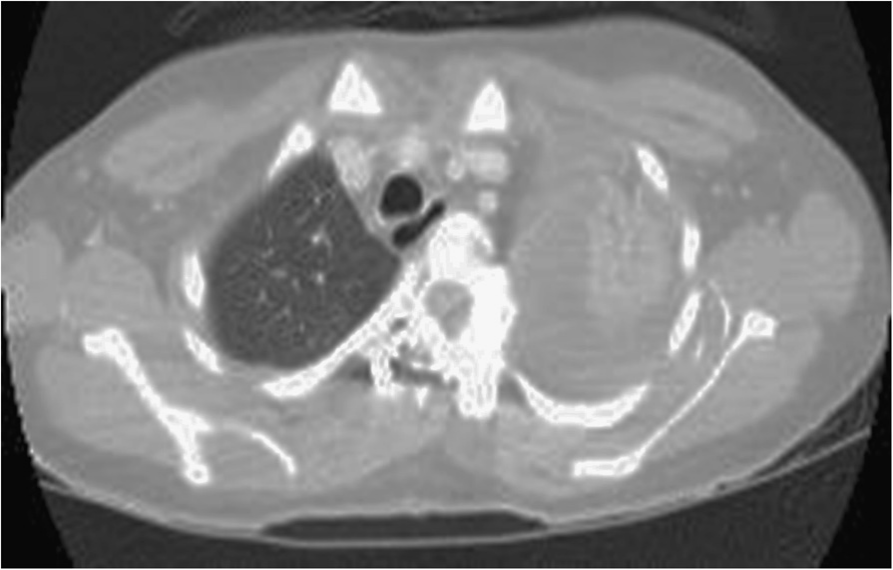
**A postoperative chest CT image.** A postoperative CT image of the chest shows a massive hemothorax in the left pleural space.

The patient’s respiratory status was stable during the surgery. After confirming 100% SpO_2_, the patient was extubated without incident. A chest x-ray taken just before extubation was normal. However, within 20 minutes after removing the tube, SpO_2_ dramatically decreased to 80% even though the patient was placed on 100% oxygen. A chest x-ray taken 25 minutes after surgery showed massive opacification in the left thorax (Figure 3). Contrast CT images of the chest revealed a massive hemothorax in the left pleural space, which was on the convex side of the upper thoracic curve (Figure 4). However, CT images did not show a definitive arterial rupture or malpositioned pedicle screw.

Since the patient’s SpO_2_ remained as low as 80-85% even with oxygen treatment, a drainage tube was inserted in the chest 110 minutes after surgery, and 400 ml blood was drained. To prevent additional hemorrhage, an intubation tube was inserted again, and then 5 cmH_2_O positive end-expiratory pressure was applied, which stopped blood drainage from the chest. The tracheal and drainage tubes were removed four days after surgery. There were no signs of dyspnea or deteriorating SpO_2_ after extubation, even though the patient was no longer on oxygen. Chest CT images taken one week after surgery showed no pleural effusion. The pulmonary function showed 88% of % vital capacity (%VC) and 95% of % predicted forced expiratory volume in one second (%FEV_1.0_) two years after surgery.

## Conclusions

Postoperative hemothorax is quite rare in posterior correction and fusion surgery for AIS. A systematic review of 1666 AIS patients who underwent posterior surgery using pedicle screw constructs identified only one hemothorax after surgery [6]. Another review of 5654 AIS patients who underwent posterior surgery also found only one hemothorax after surgery [5]. In both hemothorax cases, the hemorrhage was not substantial, and the patients were observed without further intervention.

To our knowledge, this is the first recorded case of an AIS patient who developed acute respiratory failure due to massive hemorrhage in the pleural space after posterior surgery with a pedicle screw construct, and who required the insertion of a drainage tube in the chest. Postoperative hemothorax appears to be more common in scoliosis associated with muscle dystrophy than in AIS. Modi et al. attributed this to spinal osteoporosis in patients with muscle dystrophy, which increases the risk of the vertebral body’s lateral and anterior cortical walls being breached while probing the pedicle and inserting pedicle screws [1]. They also speculated that fragility of the blood vessels, including the intercostal arteries and their branches, is a risk factor for hemothorax [1].

**Figure 5:**
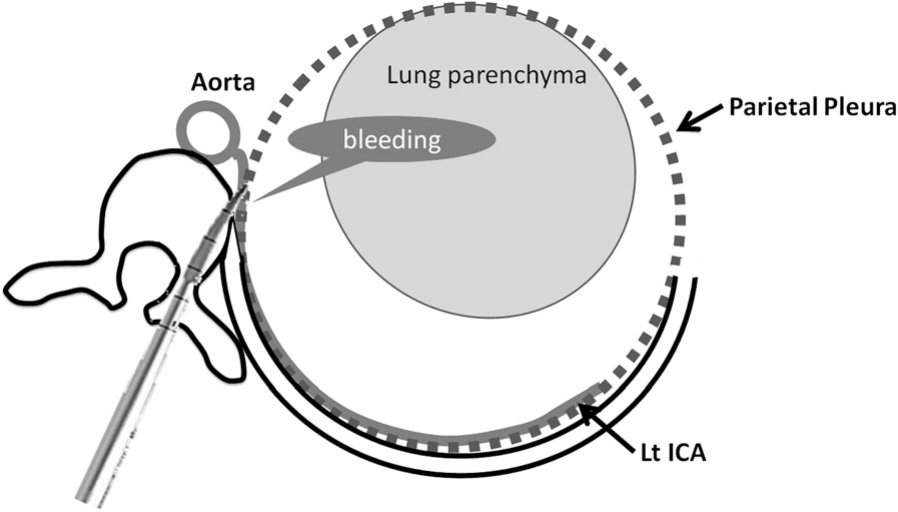
**Vascular anatomy at the pleurae.** Left Intercostal Arteries (ICAs), which branch directly from the aorta, supply the posterior part of the pleurae along each rib. Pedicle probing may have injured the left ICAs, which run near the vertebrae.

It was suspected that the hemothorax in our patient was caused by damage to a segmental vessel when probing the pedicle on the convex side of the proximal thoracic curve, since contrast CT images showed the hematoma pushing the lung away from the vertebrae in this area. Anatomically, the internal mammary arteries and their branches supply the anterior part of the pleurae, while the intercostal arteries and their branches supply the posterior part of the pleurae along each rib (Figure 5). The hemothorax may have been caused by the rupture of an intercostal artery or arterial branch. Extensive CT, MRI, and echogram evaluation after surgery did not detect any other causative factors, such as coagulopathy or pulmonary or vascular malformation.

The hemothorax appeared on a chest x-ray taken twenty minutes after extubation. Bleeding from vessels injured during pedicle probing might have been suppressed during surgery by the positive airway pressure maintained by ventilation. After extubation, the negative intrathoracic pressure introduced by spontaneous inspiration might have allowed the injured vessel to start bleeding. The positive end-expiratory pressure, 5 cmH_2_O, that we used in this case is the standard pressure used to keep the lung from collapsing at the end-expiratory phase [10]; the compressive force created by positive airway pressure successfully stopped the bleeding.

In a previous report of hemothorax after surgery to implant a cardiac pace maker, this method was reported to be effective for initially managing hemothorax if vital signs are stable [11]. If positive airway pressure does not stop the bleeding, catheter embolization or direct application of a hemostat by thoracotomy should be considered [12].

This case illustrates that placing thoracic pedicle screws carries a risk of massive hemothorax that may become apparent after extubation, and confirms the effective use of re-intubation with positive end-expiratory pressure, along with chest drainage, to treat this serious complication.

## Consent

Written informed consent was obtained from the parents of the patient for publication of this case report and any accompanying images. A copy of the written consent is available for review from the Editor-in-Chief of this journal.

## Competing interests

The authors declare that they have no competing interests.

## Authors’ contributions

YO, KW, and MM made substantial contributions to the conception and design, and the acquisition, analysis, and interpretation of data. They were also involved in drafting and revising the manuscript. NH and YT contributed to the conception and design, and performed critical revision of the manuscript. All authors read and approved the final manuscript.

## Pre-publication history

The pre-publication history for this paper can be accessed here:

http://www.biomedcentral.com/1471-2474/14/132/prepub

## References

[B1] Modi HN Suh SW Hong JY Cho JW Park JH Yang JH Treatment and complications in flaccid neuromuscular scoliosis (Duchenne muscular dystrophy and spinal muscular atrophy) with posterior-only pedicle screw instrumentation European spine journal: official publication of the European Spine Society, the European Spinal Deformity Society, and the European Section of the Cervical Spine Research Society 2010 19 3 384 393 10.1007/s00586-009-1198-z PMC2899771 19885687

[B2] Grossfeld S Winter RB Lonstein JE Denis F Leonard A Johnson L Complications of anterior spinal surgery in children J Pediatr Orthop 1997 17 1 89 95 8989708

[B3] Shapiro G Green DW Fatica NS Boachie-Adjei O Medical complications in scoliosis surgery Curr Opin Pediatr 2001 13 1 36 41 10.1097/00008480-200102000-00006 11176241

[B4] Weis JC Betz RR Clements DH 3rd Balsara RK Prevalence of perioperative complications after anterior spinal fusion for patients with idiopathic scoliosis J Spinal Disord 1997 10 5 371 375 9355051

[B5] Gautschi OP Schatlo B Schaller K Tessitore E Clinically relevant complications related to pedicle screw placement in thoracolumbar surgery and their management: a literature review of 35,630 pedicle screws Neurosurg Focus 2011 31 4 E8 10.3171/2011.7.FOCUS11168 21961871

[B6] Hicks JM Singla A Shen FH Arlet V Complications of pedicle screw fixation in scoliosis surgery: a systematic review Spine 2010 35 11 E465 E470 10.1097/BRS.0b013e3181d1021a 20473117

[B7] Levine DS Dugas JR Tarantino SJ Boachie-Adjei O Chance fracture after pedicle screw fixation. A case report Spine 1998 23 3 382 385 discussion 386 10.1097/00007632-199802010-00019 9507630

[B8] Fu KM Smith JS Polly DW Ames CP Berven SH Perra JH Glassman SD McCarthy RE Knapp DR Shaffrey CI Morbidity and mortality associated with spinal surgery in children: a review of the scoliosis research society morbidity and mortality database J Neurosurg Pediatr 2011 7 1 37 41 10.3171/2010.10.PEDS10212 21194285

[B9] Watanabe K Matsumoto M Tsuji T Ishii K Takaishi H Nakamura M Toyama Y Chiba K Ball tip technique for thoracic pedicle screw placement in patients with adolescent idiopathic scoliosis J Neurosurg Spine 2010 13 2 246 252 10.3171/2010.3.SPINE09497 20672962

[B10] Brower RG Lanken PN MacIntyre N Matthay MA Morris A Ancukiewicz M Schoenfeld D Thompson BT Higher versus lower positive end-expiratory pressures in patients with the acute respiratory distress syndrome N Engl J Med 2004 351 4 327 336 15269312 10.1056/NEJMoa032193

[B11] Lai CH Chen JY Wu HY Wen JS Yang YJ Successful conservative management with positive end-expiratory pressure for massive haemothorax complicating pacemaker implantation Resuscitation 2007 75 1 189 191 10.1016/j.resuscitation.2007.03.004 17467866

[B12] Hunt PA Greaves I Owens WA Emergency thoracotomy in thoracic trauma-a review Injury 2006 37 1 1 19 10.1016/j.injury.2005.02.014 16410079

